# Usefulness of the additional contrast agents to identify offending nerve root in selective nerve root block

**DOI:** 10.1097/MD.0000000000025131

**Published:** 2022-02-04

**Authors:** Sangbong Ko, Junho Nam, Jaejun Lee

**Affiliations:** Department of Orthopedic Surgery, College of Medicine, Daegu Catholic University, Daegu, Korea.

**Keywords:** contrast agent, functional outcome, quality of life, selective nerve root block

## Abstract

It is hypothesized that if it is confirmed that the pain caused by the injection needle coincides with the lower leg radiating pain(LLRP) that the patient mainly complains of, then the contrast agents may be used less. This study aims to understand if the identification of lancinating identical pain in the procedure could replace the use of contrast agents that causes additional pain provocation using control arm of randomized clinical trial.

This retrospective study included 165 patients who met exclusion criteria from among patients who underwent Selective nerve root block for the treatment of LLRP. With the identical and lancinating pain confirmed in the same site of the patient, consistent with that of the original symptom, the subjects were divided into 2 groups: 1 without contrast injection (Non-Dye [ND] group; 57 patients) and the other with contrast injection (Dye [D] group; 108 patients). The degree of LLRP in the 2 groups was evaluated using visual analog scale (VAS) before injection, 2, 6, and 12 weeks after injection. Functional outcomes were measured using Owestry Disability Index and Rolland-Morris Disability Questionnair, whereas quality of life was measured using Physical component score and Mental component score of Short Form 36 (SF-36) before injection and 3 months after injection.

There was no statistically significant difference in the LLRP severity in both groups at all times and no statistical difference in the degree of VAS improvement relative to the before-injection VAS value between the 2 groups at 2 and 6 weeks after injection (all *P* > .05). At 12 weeks after injection, there was a statistically significant difference, but they were below Minimum Clinical Important Difference, bearing little clinical implications. There was no statistically significant difference between the 2 groups in Owestry Disability Index, Rolland-Morris Disability Questionnair, SF-36 Physical component score, and SF-36 Mental component score at every interval (all *P* > .05).

Instead of contrast agent injections that have been used for accurate nerve root identification during Selective Nerve Root Block, the method of merely checking if the needle-induced pain under fluoroscopic imaging is consistent with the LLRP that the patient predominantly experiences shows the same effect in the patient's pain control and functional outcome.

## 1 Introduction

Lower leg radiating pain (LLRP) usually occurs in the hip or buttock areas. These pains are radiated to the thighs, calves, ankle joints and soles, and these lancinating feelings of stabbing, burning, electrifying, dull, or worms crawling are very sensitive symptoms occurring in nerve root irritated symptoms. To alleviate this radiating pain, manual therapy, physical therapy, massage, and various drug treatments have been presented, and among them, anti-inflammatory agents, muscle relaxants, and calcium channel blockers (gabapentin, pregabalin) have been under investigation in a number of studies. Recently, epidural injection therapy and Selective Nerve Root Block (SNRB) are commonly practiced as minimally invasive treatments. SNRB is a method for alleviating pain by injecting glucocorticoids and local anesthetics into the compressed nerve roots that cause radiating pain. Disagreement persists over the ultimate therapeutic effect of SNRB but the dominating opinion among researchers is that it is effective in the short term.^[1,2]^

However, various complications may occur during this SNRB. Among these are adverse reactions to gluococorticoid and radiation exposure, as well as various problems caused by the contrast agents used to identify the injection site.^[3–5]^ Although less common, there is a possibility of anaphylactic shock due to contrast agents. It is independent of the method of administration and dosage, difficult to predict if there is no history of anaphylactic shock after the use of contrast agents, and sometimes very fatal to the patient. In addition, while injecting contrast agents may give patients additional LLRP, additional radiation exposure, or delay in procedure time, care should be taken when they are used in patients with renal disease. Therefore, it is hypothesized that if it is confirmed that the pain caused by the injection needle coincides with the radiating pain that the patient mainly complains of, then the contrast agents that cause additional side effects may be used less.

The purpose of this study was to understand if the identification of lancinating identical pain in the procedure could replace the use of contrast agents by dividing the patients into 2 groups, 1 with contrast agent injection to ensure that the needle tip was located in the nerve root to be blocked and the other without contrast agent injection but with lancinating identical pain imposed in the same site and compare the 2 groups in terms of improvement in pain and functional results.

## 2 Materials and methods

### 2.1 Patient population

This trial was approved by the institutional review board (approval number: CR-20-058) of our institution and conducted in accordance with the declaration of Helsinki. The subject pool was a total of 655 patients with radiating pain of 5 or higher on the visual analog scale (VAS) who underwent SNRB for therapeutic purposes between January 2015 and November 2019. Lesions consistent with symptoms in those patients were identified using magnetic resonance imaging and 165 patients who met all exclusion criteria were analyzed. Patients who were pregnant, with secondary interests (industrial accidents, auto insurance, etc.), with serious comorbidities, who could not be followed up for more than 3 months after injection for personal reasons, with contraindications to the drug, under an intervention study during the study period, and with cancer pain due to primary or metastatic cancer in the spine were excluded (Table 1). The degree of LLRP was measured using VAS during the initial outpatient visit. This study was retrospective study on data collected prospectively.

**Table 1:** Inclusion and exclusion criteria.

Inclusion criteria	
1	LANSS score >7
2	Radiating pain VAS ≥5
3	Agreement for participating
4	Foraminal stenosis in MRI
5	Symptomatic relief of over 70% immediately after injection
Exclusion criteria	
1	Pregnant woman
2	Patients with secondary gains(e.x., worker's compensation etc)
3	Significant comorbidity
4	Follow up loss due to move, personal issue etc.
5	Patients contraindicated to medications used in SNRB
6	Patients participated in other studies during or right before the study
7	Patients with cancer pain either due to primary or metastatic cancer
8	Acute radiculopathy to the lower extremities due to herniated disc
9	Patients who cannot understand the questions of the questionnaire

LANSS Score = the leeds assessment of neuropathic symptoms and signs, MRI = magnetic resonance imaging, SNRB = selective nerve root block, VAS = visual analogue scale.

### 2.2 Procedures for conducting SNRB

**Figure 1:**
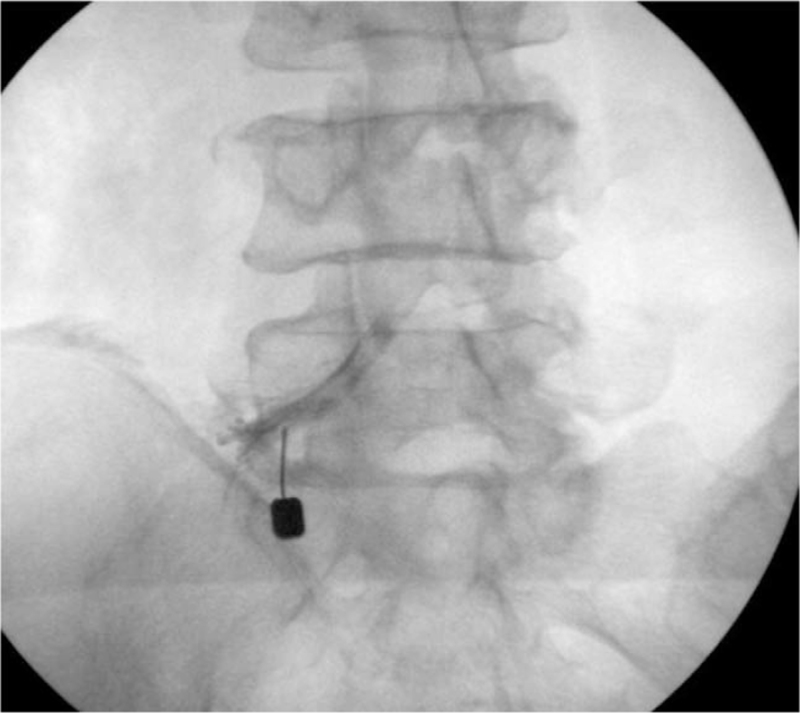
Contrast media was injected to confirm the culprit nerve root.

All SNRBs were performed in the outpatient setting with no premedication. The patients were placed in a prone position on the operating table, and standardized sterilization procedures were carried out. Oblique plain radiographs were acquired to confirm injection sites. Local anesthesia was administered (1% lidocaine) followed by injection of medication via a 23-gauge spinal needle under fluoroscopic guidance. A spinal needle was advanced at a safe triangle in the spinal root site. Patients were randomly divided into 2 groups. Random allocation of treatment regiments using permuted block randomization method was performed by another doctor who was not involved in treatment or evaluation. In 1 group (Non-Dye group: ND group), after identical and lancinating pain was confirmed, and if the location of the pain matched the site of the original symptom, medication was administered without a contrast agent, and in the other group (Dye group: D group), only twinge was confirmed as the injection needle was advanced. In such a case, contrast agent (Iohexol; Omnipaque GE Healthcare Ireland, Cork, Ireland; 300 mg/mL) was administered to confirm the injection site and location of the affected spinal nerve root (Fig. 1). If the confirmed injection site was consistent with the site of symptoms and Magnetic Resonance Imaging findings, then medications were injected into the nerve root via the same route. For injection, a total of 3 mL of the mixture containing 1 mL each of triamcinolone, 0.25% bupivacaine, and normal saline was prepared, and the maximum injection amount was approximately 1.5 mL, with 0.5 to 1.5 mL injected in most cases.

### 2.3 Outcome measurements

The severity of LLRP was assessed using VAS on a scale of 0 (no pain) to 10 (the worst pain imaginable). The degree of pain was assessed at 2, 6, and 12 weeks after injection. The spine-related functional outcome was assessed using the Oswestry Disability Index (ODI) and Roland–Morris Disability Questionnaire (RMDQ) before injection and 3 months after injection, whereas quality of life was measured using SF-36, which was divided into mental component score (MCS) and physical component score (PCS), before injection and 3 months after injection.

### 2.4 Statistical analysis

One-way analysis of variance, correlation analysis, and repeated-measures single-factor analysis methods were used. Fisher exact test for the distribution of gender and injection nerve roots were used. Data are presented as mean ± standard deviation. The IBM Statistical Package for the Social Sciences version 19.0 for Windows (Statistical Package for the Social Sciences Inc., Chicago, IL) was used for all statistical analyses. A *P* value of ≤.05 was considered statistically significant.

## 3 Results

### 3.1 Epidemiological results

Out of 165 patients (61 men, 104 women) in total, 57 (24 men, 33 women) were in ND group, and 108 (37 men, 71 women) in D group. There was no statistical significance for the sex ratio of each group (*P* = .309). The average age was 68.46 ± 11.21 years old in total, 69.70 ± 9.61 in ND group, and 67.81 ± 11.92 in D group, which was not statistically significant (*P* = .305). There were 9 patients in the ND group and 13 patients in the D group who had decompression surgery following no improvement after SNRB. Nerve root sites included 7 L3 roots, 24 L4 roots, and 26 L5 roots in the ND group and 15 L3 roots, 51 L4 roots, 40 L5 roots, and 2 S1 roots in D group (Table 2).

**Table 2:** Epidemiological results of all populations.

Variables	Group ND	Group D	*P* value
Gender			
Male	24 (43%)	37 (34%)	.309
Female	33 (57%)	71 (66%)	
Age (Year-old)	69.70 ± 9.61	67.81 ± 11.92	.305
Level			
L3 root	7	15	
L4 root	24	51	
L5 root	26	40	
S1 root	0	2	

### 3.2 Results of LLRP

The VAS before injection was 7.35 ± 1.73 in the ND group and was 7.17 ± 1.70 in the D group. The VAS at 2 weeks after injection was 3.07 ± 2.34 in the ND group and 3.40 ± 2.29 in the D group. The VAS at 6 weeks after injection was 3.47 ± 2.60 in the ND group and 3.83 ± 2.40 in the D group. The VAS at 12 weeks after injection was 3.19 ± 2.99 in the ND group and 3.81 ± 2.27 in the D group. There was no statistically significant difference in VAS between ND group and D group before injection (*P* = .515), 2 weeks after injection (*P* = .389), 6 weeks after injection (*P* = .378), and 12 weeks after injection (*P* = .116).

There was also no statistically significant difference in improvement in VAS scores in the 2 weeks and 6 weeks after injection in the 2 groups, compared with those before injection (*P* = .167, *P* = .292). At 12 weeks after injection, the group difference was statistically significant (4.37 ± 2.81 in Group ND and 3.48 ± 2.43 in Group D) (*P* = .037), but these scores were not above 5 in Minimum Clinical Important Difference of Parker et al,^[6]^ bearing no clinical implications (Table 3).

**Table 3:** Serial VAS change and between-group difference.

	Group ND	Group D	*P* value
Initial (Preinjection)	7.35 ± 1.73	7.17 ± 1.70	.515
2 wks after injection	3.07 ± 2.34	3.40 ± 2.29	.389
6 wks after injection	3.47 ± 2.60	3.83 ± 2.40	.378
12 wks after injection	3.19 ± 2.99	3.81 ± 2.27	.116
VAS improvement, compared to initial VAS			
Preinjection – 2 wks	4.30 ± 2.55	3.79 ± 2.08	.167
Preinjection – 6 wks	3.91 ± 2.69	3.50 ± 2.20	.292
Preinjection – 12 wks	4.37 ± 2.81	3.48 ± 2.43	.037^∗^

VAS = visual analogue scale.

∗ *P* < .05.

### 3.3 Results of functional outcomes

Before injection, ODI was 21.82 ± 8.92, RMDQ 11.30 ± 6.19, SF-36 PCS 27.76 ± 14.38, and SF-36 MCS 44.42 ± 20.91 in Group ND, whereas ODI was 23.24 ± 9.16, RMDQ 11.40 ± 6.81, SF-36 PCS 26.33 ± 17.59, and SF-36 MCS 39.32 ± 20.15 in Group D. At 3 months after injection, ODI was 16.00 ± 9.21, RMDQ 8.54 ± 6.18, SF-36 PCS 41.50 ± 23.73, and SF-36 MCS 51.41 ± 22.40 in ND group, and ODI was 17.93 ± 10.20, RMDQ 9.07 ± 9.24, SF-36 PCS 41.78 ± 22.41, and SF-36 MCS 50.69 ± 20.85 in D group. There was no statistically significant difference in ODI, RMDQ, SF-36 PCS, and SF-36 MCS between ND group and D group at both the before injection and 12 weeks after injection (all *P* > .05). There was no statistically significant difference in the improvement rate of ODI, RMDQ, SF-36 PCS, and SF-36 MCS at 3 months after injection between the 2 groups, compared with that before injection (*P* > .05) (Table 4).

**Table 4:** Results of functional outcome and between-group difference.

	Group ND	Group D	*P* value
Initial			
ODI	21.82 ± 8.92	23.24 ± 9.16	.349
RMDQ	11.30 ± 6.19	11.40 ± 6.81	.928
SF-36 PCS	27.76 ± 14.38	26.33 ± 17.59	.611
SF-36 MCS	44.42 ± 20.91	39.32 ± 20.15	.145
12 wks			
ODI	16.00 ± 9.21	17.93 ± 10.20	.240
RMDQ	8.54 ± 6.18	9.07 ± 9.24	.704
SF-36 PCS	41.50 ± 23.73	41.78 ± 22.41	.944
SF-36 MCS	51.41 ± 22.40	50.69 ± 20.85	.844
Improvement, compared to initial			
ODI	5.72 ± 10.58	5.49 ± 12.90	.909
RMDQ	2.61 ± 6.97	2.11 ± 11.24	.760
SF-36 PCS	10.83 ± 25.21	15.52 ± 22.05	.221
SF-36 MCS	5.72 ± 31.25	11.88 ± 25.76	.180

ODI = oswestry disability index, RMDQ = rolland-morris disability questionnaire, SF-36 MCS = short form 36 mental component score, SF-36 PCS = short form 36 physical component score.

## 4 Discussion

SNRB began being performed in 1971 by Macnab et al^[7]^ In this procedure a needle tip is inserted under fluoroscopic guidance into the root sleeve of the nerve root and then, the exact site is confirmed using radio-opaque dyes and with induced pain in the patient, and injection is made to alleviate the radiating pain. Since then, various options for nerve root identification have been used, including computed tomography, ultrasound imaging, and electrostimulation,^[8–12]^ but until now, the method by Macnab et al has been used most widely.^[13]^ Each radiological finding, however, included the use of a contrast agent, even though in a small amount, for nerve root identification. Pfirrmann et al^[14]^ and Irwin et al^[15]^ classified the anatomical position of the contrast agent into intraneural, extraneural, and perineural to judge the tip of the needle and analyzed the effect of the injection. After confirming the nerve root by inducing LLRP due to the progression of the needle tip under the initial radiographic image guidance, pulling the needle slightly back and then injecting 0.5 mL of contrast media to confirm the correct level, they injected adrenocortical hormone and a local anesthetic. Although the needle tip is withdrawn slightly after pain provocation, the patient must experience additional pain provocation when contrast media are injected. The correct level is confirmed 3 times: fluoroscopic imaging during SNRB, pain provocation in the lower extremity by the needle tip as the needle progresses, and radiological imaging using contrast media. Given that the injection site is determined using fluoroscopic imaging, pain provocation by a needle tip as the needle progresses, and determining whether this pain is identical to the pain in the lesion consistent with the area of the patient's lower extremity pain, this study aimed to investigate whether circumventing the injection site identification by additional contrast media was possible.

In a study involving 283 patients with nerve root block of the cervical, thoracic, and lumbar spine, Mallinson et al^[16]^ reported that their analgesic outcome was independent of the use of contrast agents. However, the authors confirmed that the location of the injection needle was confirmed by the operator's reassurance and the fact that it was not the intravascular needle placement and did not describe the method of reassurance accurately. In addition, it was suggested that the reason for no difference in their results was due to local diffusion or systemic effect of the injectant, but a clear causal relationship was not presented. Mallinson et al^[16]^ and Pfirrmann et al^[14]^ claim that the pain provocation caused by the contrast agent indicates that the contrast agent means intraepineural injection and has little to do with the patient's functional outcome. After all, they argued that not only the contrast agent used in SNRB but also the pain provocation caused by the contrast agent itself is independent of the patient's functional outcome and the contrast agent is used only for re-identification of the nerve roots that cause the radiating pain in the patient's lower extremity.

Contrast agents used in SNRB can lead to fatal adverse effects due to anaphylactic shocks in some patients and may cause acute renal failure in vascular disease patients, diabetics, and elderly people with renal disease. There may also be a risk of unpleasant sensation to the patient due to lower extremity pain caused by injection of contrast medium once again after radiating pain is induced in the extremity pain through the injection needle, and direct intraepineural injection of the contrast agent may lead to mechanical impairment. In addition, extended procedure time, additional radiation exposure, and added cost burden due to the insertion of the contrast agent may also be a problem.

The limitations of this study are as follows: First, there was a lack of data on side effect of corticosteroid which occurred actually during this study. Second, only the results of improvement of VAS, functional outcome and quality of life were obtained. Third, a relatively short follow-up period of 3 months is one of the limitations. Forth, the number of patients in each group was too small to analyze the mechanism and related factors, although this was a randomized method.

## 5 Conclusion

During SNRB, fluoroscopic imaging, pain provocation caused by the tip of the needle, and whether the radiating pain induced in the lower extremity matches the patient's chief complaint are sufficient to identify the nerve roots correctly, which can lead to reduced use of contrast agents. It reduces rare fatal hypersensitivity shocks associated with the dye and acute renal failure in high-risk patients, delayed procedure time following dye injection, additional radiation exposure, and unpleasant sensation to the patient by contrast injection, as well as mechanical impairment by intraepineural injection of additional contrast medium.

## Author contributions

**Conceptualization:** Sangbong Ko.

**Data curation:** Sangbong Ko, Junho Nam.

**Formal analysis:** Sangbong Ko.

**Funding acquisition:** Sangbong Ko.

**Investigation:** Sangbong Ko.

**Methodology:** Sangbong Ko.

**Project administration:** Sangbong Ko.

**Resources:** Sangbong Ko.

**Software:** Sangbong Ko, Junho Nam.

**Supervision:** Sangbong Ko.

**Validation:** Sangbong Ko.

**Visualization:** Sangbong Ko.

**Writing – original draft:** Sangbong Ko.

**Writing – review & editing:** Sangbong Ko, Junho Nam, Jaejun Lee.
